# Inequities in referrals to a breast cancer risk assessment and prevention clinic: a mixed methods study

**DOI:** 10.1186/s12875-023-02126-1

**Published:** 2023-08-25

**Authors:** Claire B. King, Brittany L. Bychkovsky, Erica T. Warner, Tari A. King, Rachel A. Freedman, Elizabeth A. Mittendorf, Fisher Katlin, Anna Revette, Danielle M. Crookes, Neil Maniar, Lydia E. Pace

**Affiliations:** 1 Comprehensive Breast Health Center, Brigham and Women’s Hospital, Boston, MA USA; 2 Division of Breast Surgery, Department of Surgery, Brigham and Women’s Hospital, Boston, MA USA; 3 Division of Cancer Genetics and Prevention, Dana-Farber Cancer Institute, Boston, MA USA; 4 Department of Medical Oncology, Dana-Farber Cancer Institute, Boston, MA USA; 5 Breast Oncology Program, Dana-Farber Brigham Cancer Center, Boston, MA USA; 6 Harvard Medical School, Boston, MA USA; 7 Massachusetts General Hospital, Boston, MA USA; 8 Division of Population Science, Dana-Farber Cancer Institute, Boston, MA USA; 9 Harvard T.H. Chan School of Public Health, Boston, MA USA; 10 Department of Health Sciences, Northeastern University, Boston, MA USA; 11 Department of Sociology and Anthropology, Northeastern University, Boston, MA USA; 12 Division of Women’s Health, Brigham and Women’s Hospital, Boston, MA USA

**Keywords:** Health equity, Breast cancer, Referrals, Screening, Prevention, Risk evaluation

## Abstract

**Background:**

Inequitable access to personalized breast cancer screening and prevention may compound racial and ethnic disparities in outcomes. The Breast Cancer Personalized Risk Assessment, Education and Prevention (B-PREP) program, located within the Brigham and Women’s Hospital (BWH) Comprehensive Breast Health Center (BHC), provides care to patients at high risk for developing breast cancer. We sought to characterize the differences between BWH primary care patients referred specifically to B-PREP for risk evaluation and those referred to the BHC for benign breast conditions. Through interviews with primary care clinicians, we sought to explore contributors to potentially inequitable B-PREP referral patterns.

**Methods:**

We used electronic health record data and the B-PREP clinical database to identify patients referred by primary care clinicians to the BHC or B-PREP between 2017 and 2020. We examined associations with likelihood of referral to B-PREP for risk assessment. Semi-structured interviews were conducted with nine primary care clinicians from six clinics to explore referral patterns.

**Results:**

Of 1789 patients, 78.0% were referred for benign breast conditions, and 21.5% for risk assessment. In multivariable analyses, Black individuals were less likely to be referred for risk than for benign conditions (OR 0.38, 95% CI:0.23–0.63) as were those with Medicaid/Medicare (OR 0.72, 95% CI:0.53–0.98; OR 0.52, 95% CI:0.27–0.99) and those whose preferred language was not English (OR 0.26, 95% CI:0.12–0.57). Interviewed clinicians described inconsistent approaches to risk assessment and variable B-PREP awareness.

**Conclusions:**

In this single-site evaluation, among individuals referred by primary care clinicians for specialized breast care, Black, publicly-insured patients, and those whose preferred language was not English were less likely to be referred for risk assessment. Larger studies are needed to confirm these findings. Interventions to standardize breast cancer risk assessment in primary care may improve equity.

**Supplementary Information:**

The online version contains supplementary material available at 10.1186/s12875-023-02126-1.

## Introduction

Inequities in breast cancer care span the continuum from prevention to screening to treatment [1–5]. Non-English speaking, Black, and Hispanic women are more often diagnosed with late-stage breast cancer compared to White women [2, 6–10] and Black women in the United States have 40% higher breast cancer mortality. Although current standards require that screening and preventive interventions be tailored to individuals’ risk level, [11–13] a limited body of evidence suggests that Black and Hispanic women are less likely to receive breast cancer risk assessment, genetic testing, and intensified screening [8–10, 14–16]. Gaps in risk assessment and individualized care may contribute to disparities in stage at diagnosis and outcome, and are critical to understand and address as personalized approaches to breast cancer screening and prevention evolve.

The Breast Cancer Personalized Risk Assessment, Education, and Prevention (B-PREP) program at Brigham and Women’s Hospital (BWH) helps individuals determine and understand their breast cancer risk, including counseling on enhanced screening, risk-reducing therapies including chemoprevention, and the role of genetic testing. Patients access B-PREP services via one of two pathways (Supplemental Fig. 1, Additional File 1). All individuals who are referred to the BWH Comprehensive Breast Health Center (BHC) for benign breast conditions (e.g. breast symptoms) are asked to complete a customized survey adapted from Hughes RiskApps and are internally referred to B-PREP if identified to have an elevated risk of developing breast cancer [17]. Alternatively, patients can be directly referred to B-PREP from primary care or other clinicians for risk assessment. If referred from primary care, referrals are be placed in the electronic medical record using the same process for both BHC and B-PREP.

**Fig. 1:**
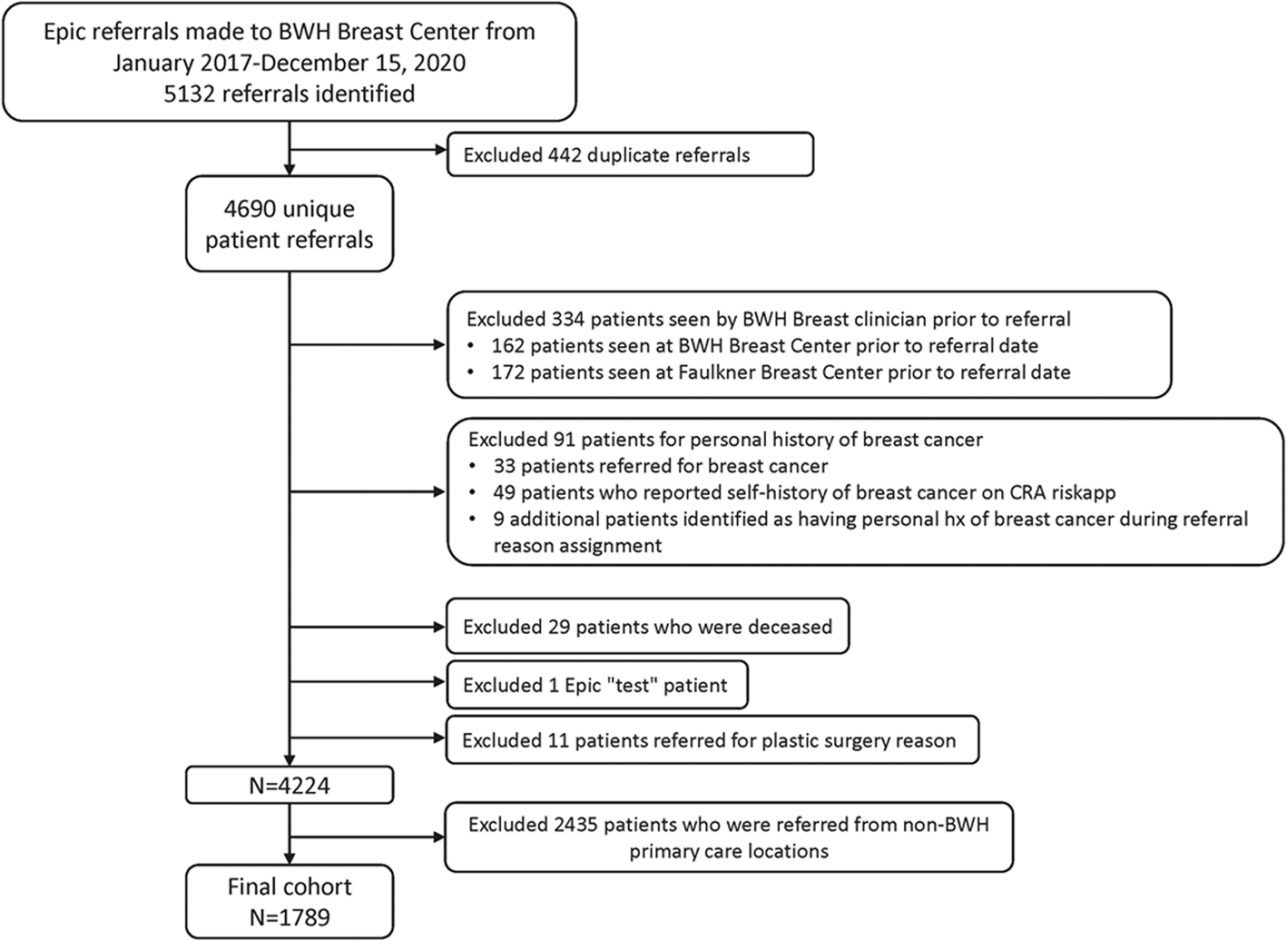
Consort diagram

Early program data suggested that patients cared for in B-PREP are disproportionately White, [17] raising concern for missed opportunities to provide breast cancer preventive and screening services to groups that often experience delayed diagnoses and adverse outcomes. However, patients are referred to B-PREP by clinicians from all over Massachusetts and neighboring states, and whether the relatively low racial and ethnic diversity simply reflected the demographics of referring practices was not clear. To further understand this issue, we conducted a mixed methods assessment to examine whether there was evidence of inequitable referral patterns specifically from BWH primary care clinics, which overall serve a more racially and ethnically diverse population than B-PREP. We sought to quantitatively compare the characteristics of those referred from BWH primary care to the BHC for non-specific breast concerns versus those referred directly to B-PREP for risk assessment. We then interviewed primary care clinicians to identify factors that influence B-PREP referrals. Although patients referred to the BHC may still access B-PREP services if they are identified as elevated risk, lower rates of referral for risk assessment among patients from historically marginalized groups could suggest missed opportunities for those patients who are not referred to BHC and inform interventions to improve care quality and equity.

## Methods

### Study design

We used an explanatory sequential design for this mixed methods study, first collecting quantitative data collection and using subsequent clinician interviews to further explore and understand these findings [18].

### Setting

BWH is a large academic medical center in Boston, MA with 18 affiliated primary care clinics including community health centers, urban academic clinics located at or near the main campus and suburban sites. B-PREP was established in 2017 within the larger BHC, which is located on the BWH main campus.

### Quantitative study cohort

Referrals made to the BHC or B-PREP from January 1, 2017, through December 15, 2020, were obtained from the electronic health record (EHR). Information included primary care clinician and practice, insurance, patient race and ethnicity, zip code, preferred language, reason for referral including associated free text, and ICD-10 diagnostic code for the referral. After duplicate referrals were removed, there were 4690 patient referrals (Fig. 1). We subsequently excluded patients with a current diagnosis or personal history of breast cancer, and those who had care at the BHC prior to their referral date. We then identified patients referred by BWH primary care sites to obtain a final cohort of 1789 unique patient referrals (Fig. 1). Of note, patients were included in our dataset even if they ultimately were not seen in the BHC or B-PREP.

We identified the reason for referral using categorial options from the EHR, free text associated with the referral, and the entered ICD-10 diagnostic code (Supplemental Fig. 2). Associated terms (Referral Reason Classification, Additional file 1) were used to classify patients as referred for risk assessment or for benign conditions, including those in need of surgical evaluation. Our outcome variable was referral for risk versus referral for benign conditions. Covariates were patient age (<40, 40–59, ≥ 60), race and ethnicity (categorized as non-Hispanic White, non-Hispanic Black, Hispanic, Asian American/Pacific Islander, other, unknown), preferred language (categorized as English versus non-English), insurance type (categorized as Medicaid, Medicare, commercial, other), and type of referring primary care practice (grouped by main campus, community health center, or suburban site). For patients for whom a zip code was available, we obtained their corresponding Social Vulnerability Index, a measure developed by the Centers for Disease Control that uses Census tract-level socioeconomic and demographic characteristics to estimate community resilience [19]. We additionally abstracted patients’ breast cancer risk level, based on the Gail and Tyrer-Cuzick risk calculators completed upon patients’ clinic intake in the BHC or B-PREP. We identified patients as “high-risk” if they either had a 5-year modified Gail risk score of ≥ 1.67% for women between age 35–59, a 5-year modified Gail risk score of > 5.5% for women age ≥ 60, or a Tyrer-Cuzick v.7 or v.8 lifetime risk score > 20% [20].

### Quantitative analysis

We performed descriptive analysis and utilized Chi-Square tests and multivariable logistic regression to examine unadjusted and adjusted associations with likelihood of referral to B-PREP for risk assessment. We conducted a sensitivity analysis in which we excluded patients with unknown risk scores from the multivariable model.

### Qualitative methods

For this mixed methods study, we used an explanatory sequential design, allowing our quantitative findings to inform our qualitative approach [18]. We developed an interview guide based on our quantitative findings and on our conceptual model (Supplemental Fig. 3), focusing on breast cancer risk assessment practices in primary care as well as clinicians’ awareness and utilization of the B-PREP program. We also asked about clinicians’ perceptions of contributors to the observed racial/ethnic differences in referral patterns. We utilized a purposive sample of clinicians across several BWH primary care sites including the BWH main campus, two community health centers, and suburban sites. Primary care clinicians were selected to capture a range of facility types and patient populations, as well as a range of levels of experience. Clinicians were invited to participate via email. Interviews lasted approximately 30 minutes and were conducted via telephone or videoconference by one researcher (CK). We interviewed nine clinicians (eight physicians and one physician assistant) after reaching thematic saturation.

For qualitative analysis, each interview was audio-recorded, professionally transcribed, and analyzed using multi-stage thematic analysis. The code structure was iteratively developed and reviewed by four researchers (CK, BLB, LEP, AR), and incorporated both prefigured and emergent codes. Once the code structure was finalized, one researcher (CK) independently coded all transcripts using Dedoose software (Version 9.0.62, Los Angeles, CA). After coding, analysis focused on identifying key concepts, patterns, and relationships within and between the interviews.

### Ethics

This study was performed in accordance with the Declaration of Helsinki and approved by the appropriate ethics committee. The quantitative portion of this project using data from medical records and the B-PREP risk assessment dataset was approved by the Mass General Brigham Institutional Review Board (IRB) (protocol 2018P003003). The qualitative portion of the project involving clinician interviews was reviewed by the Mass General Brigham Human Research Office, which determined that it did not meet criteria for human subject research as defined by Mass General Brigham Human Research Office policies and Health and Human Services regulations set forth in 45 CFR 46.

### Consent to participate

In the quantitative portion of the project, the requirement for informed consent of patients whose charts were reviewed was waived by the IRB. In the qualitative portion of the project involving clinician interviews, verbal informed consent was obtained from all clinician interview participants.

## Results

### Quantitative findings

Between 2017 and 2020, 1789 patients were referred from BWH-affiliated primary care clinics to the BHC for a benign breast condition or to B-PREP for risk assessment. Among these patients, 78.5% (*n* = 1404) were referred for benign conditions, and 21.5% (*n* = 385) for risk assessment. Overall, 48.5% were identified as non-Hispanic White, 22.1% Hispanic, 10.8% non-Hispanic Black, 3.9% Asian American/Pacific Islander, and 14.8% as other or unknown race and ethnicity (Table 1). Compared to our population, BWH primary care data suggest that 63% of female patients are NHW, 12% are Black or African American, 7% are Hispanic or Latinx, and 6% are Asian, suggesting our population has slightly fewer White patients, and a greater proportion of Black and Hispanic patients. By clinic site, 48.0% were referred from suburban sites, 32.3% from our main campus and 17.9% from community health centers. Among referred patients, 12.1% (216) preferred a language other than English. Mean age was 48.3 (standard deviation 14.56). Zip code and Social Vulnerability Index (SVI) were available for 78.4% of patients, with the remainder who had known zip code but SVI was not attainable (n = 384) or were lacking zip code information (*n* = 1).

**Table 1:** Patient demographics overall and by reason for referral

Patient Demographics	Overall (%) (*N* = 1789)	Referred to B-PREP for risk assessment (%) (*n* = 385)	Referred for benign breast issue (%) (*n* = 1404)	*P* value
**Race and Ethnicity**				
Non-Hispanic White	867 (48.5)	248 (64.4)	619 (44.1)	** < 0.001**
Non-Hispanic Black	193 (10.8)	21 (5.5)	172 (12.3)	
Hispanic	395 (22.1)	50 (13.0)	345 (24.6)	
Asian American/Pacific Islander	69 (3.9)	15 (3.9)	54 (3.8)	
Other	238 (13.3)	47 (12.2)	191 (13.6)	
Unknown	27 (1.5)	4 (1.0)	23 (1.6)	
**Age**				
Mean	48.4 (14.6)	45.6 (13.5)	49.1 (14.8)	** < 0.001**
Under 40	566 (31.6)	152 (39.5)	414 (29.5)	
40–59	802 (44.8)	157 (40.8)	645 (46.0)	
60 +	421 (23.5)	76 (19.7)	345 (24.6)	
**Risk Level**^a^				
Not High Risk	675 (37.7)	51 (13.2)	624 (44.4)	** < 0.001**
High Risk	410 (22.9)	172 (44.7)	238 (17.0)	
Unknown Risk	704 (39.4)	162 (42.1)	542 (38.6)	
**Preferred Language**				
English	1564 (87.4)	372 (96.6)	1192 (84.9)	** < 0.001**
Non-English	216 (12.1)	9 (2.3)	207 (14.7)	
Unknown	9 (0.5)	4 (1.0)	5 (0.4)	
**Insurance**				
Medicaid	600 (33.5)	82 (21.3)	518 (36.9)	** < 0.001**
Medicare	82 (4.6)	13 (3.4)	69 (4.9)	
Commercial	1080 (60.4)	286 (74.3)	794 (56.6)	
Other	18 (1.0)	3 (0.8)	15 (1.1)	
Unknown	9 (0.5)	1 (0.3)	8 (0.6)	
**Primary Care Site**				
Main Campus	577 (32.3)	131 (34.0)	446 (31.8)	**0.003**
Community Health Center	321 (17.9)	44 (11.4)	277 (19.7)	
Suburban	858 (48.0)	202 (52.5)	656 (46.7)	
Other	33 (1.8)	8 (2.1)	25 (1.8)	
**Social vulnerability index (SVI); *n* = 1,404**^b^				
Median (Interquartile range)	0.17 (0.31)	0.15 (0.27)	0.17 (0.34)	**0.001**

^a^High-risk was defined as patients having a 5-year modified Gail risk score of ≥ 1.67 for women between age 35–59, a 5-year modified Gail risk score of ≥ 5.5% for women age ≥ 60, [21] or a Tyrer-Cuzick v.7 or v.8 lifetime risk score > 20%. [22]

^b^*n* = 385 were missing zip code or an SVI could not be calculated. SVI represents the proportion of U.S. census tracts that are equally or less vulnerable to the area of interest

*B-PREP* Breast Cancer Personalized Risk Assessment, Education and Prevention program

In unadjusted analyses, the racial and ethnic distribution of patients referred to B-PREP for risk assessment was different from those referred for symptoms (*p* < 0.001; Table 1). Those referred for risk assessment were more often White (64.4% versus 44.1%) and less often Black or Hispanic. A lower proportion of those referred for risk assessment preferred a language other than English (2.3% versus 14.7%, *p* < 0.001). The insurance distribution differed among those referred for symptoms versus for risk: 74.3% of those referred for risk assessment were commercially insured versus 56.6% of those referred to BHC (*p* < 0.001). Patients from community health centers were more likely to be referred to BHC (19.7%) than for a risk assessment (11.4%) while patients from suburban sites were more prevalent among those referred for risk assessment (52.5%) compared to those referred for non-specific breast concerns (46.7%, *p* = 0.003). As expected, those referred for risk assessment had higher levels of risk.

In multivariable logistic regression analyses adjusting for patient demographics, clinic site and risk level, Black (versus White) women remained less likely to be referred to B-PREP for risk assessment (OR 0.38, 95% CI: 0.23–0.63; Table 2). Compared to patients with commercial insurance plans, patients insured by Medicaid were also less likely to be referred to B-PREP for risk assessment (OR 0.72, 95% CI: 0.53–0.98) as were those whose primary insurance was Medicare (OR 0.52, 95% CI: 0.27–0.99). Preference for a language other than English was associated with lower likelihood of being referred to B-PREP for risk assessment (OR 0.26, 95% CI: 0.12–0.57). Primary care site was not associated with referral reason in adjusted analyses.

**Table 2:** Multivariable logistic regression analysis examining factors associated with referral for breast cancer risk assessment

Patient Demographics	Odds Ratio (95% CI)	*P* value
**Race and Ethnicity**		
Non-Hispanic White	Ref	
Non-Hispanic Black	**0.38 (0.23–0.63)**	** < 0.001**
Hispanic	0.71 (0.47–1.08)	0.11
Asian American/Pacific Islander	0.82 (0.43–1.54)	0.53
Other	0.72 (0.49–1.04)	0.08
Unknown	0.59 (0.19–1.79)	0.35
**Age**		
Under 40	Ref	
40–59	**0.63 (0.48–0.84)**	**0.001**
60 +	0.73 (0.52–1.04)	0.08
**Preferred Language**		
English	Ref	
Non-English	**0.26 (0.12–0.56)**	** < 0.001**
Risk Level^*^		
Not High Risk	Ref	
High Risk	**7.14 (4.99–10.23)**	** < 0.001**
Unknown Risk	**3.35 (2.38–4.72)**	** < 0.001**
**Insurance**		
Commercial	Ref	
Medicaid	**0.72 (0.53–0.98)**	**0.04**
Medicare	**0.52 (0.7–0.99)**	**0.047**
Other	0.53 (017–1.66)	0.33
**Primary Care Site**		
Suburban	Ref	
Main Campus	1.25 (0.94–1.65)	0.13
Community Health Center	1.17 (0.76–1.82)	0.48
Other	1.15 (0.49–2.71)	0.75

^*^High-risk was defined as patients having a 5-year modified Gail risk score of ≥ 1.67 for women between age 35–59, a 5-year modified Gail risk score of ≥ 5.5% for women age ≥ 60, [21] or a Tyrer-Cuzick v.7 or v.8 lifetime risk score > 20%. [22]

Because of the prevalence of missing zip code and/or SVI, we did not include SVI in our main model. However, in a sensitivity analysis including only the 1,404 patients with available SVI in our multivariable model, SVI quartile was not significantly associated with likelihood of referral for risk assessment. Including SVI in the model also did not alter the associations noted in the main model (Supplemental Table 1, Additional file 1).

In the sensitivity analysis excluding patients with unknown risk scores, the direction of the associations between race/ethnicity, insurance, and language with referrals for risk did not change (Supplemental Table 2).

### Qualitative findings

Among the invited primary care clinicians, 91% (10 of 11) agreed to participate in the study (one clinician invited never responded to email invitations), and 82% (9 of 11) of those approached completed their interview. Of the nine clinicians interviewed, five practiced at a main hospital campus clinic, one at a suburban clinic, and three at a community health center. All participants identified as female and 2/3 were White (Supplemental Table 3, Additional file 1). We identified the following themes:

1. Risk assessment practices varied across primary care clinicians and were not systematic or comprehensive

**Table 3:** Example quotations from interviewed primary care clinicians describing risk assessment practices and awareness/approach to B-PREP referrals

Approach to risk assessment	Example quotes
Focus on family history	“I always take a family history and I always update the family history once a year…that’s kind of the red flag for me…into whether they need further evaluation in terms of their risk.” “I ask every single patient when I'm scheduling a mammogram about their family history, if I have not done so already.” “I generally ask a patient history: their own history, family history, menarche….There’s not really anything else.” “I tend to focus on family history in order to assess risk and I usually have a brief discussion about USPSTF guidelines (and) the ACS guidelines…then we use that plus our gestalt of that patient’s risk to make a plan.”
Timing and frequency of risk discussions	“If they don't have a strong family history, then I just start the discussion at age 40.” “I try to talk about (breast cancer risk) with every patient, either when they turn 40 or – and then I update it at age 45 and age 50.” “I start at age 40 because some guidelines recommend women start screening at age 40.” “At age 40, that’s when I have a more directive conversation (about risk), and I might be more inclined to do a formal assessment if somebody is having trouble deciding whether they want to get a mammogram or not.” “(I discuss risk) usually every year, at least to some extent. It’s probably not a long conversation, but at least I will check to see have they had a mammogram. Do they need one? Has anything changed?”
Use of risk assessment tools	“There might be some (tools) that I should be using but I don't use. I'm certainly aware of other risk factors, but I don't really use (the tools) in changing what I would do, you know?” “I either use the BRCA risk tool from – on the NIH website, or the breast cancer surveillance consortium risk tool. Rarely, if a patient has a complicated family history, I might use the Tyrer-Cuzick model.” “I know there are some online tools, and I haven’t accessed them recently, so I can’t tell you what they are. But I'd do a search if I needed to or felt like I needed to. But I haven’t – I probably should.”
Time constraints	“I don’t (use breast cancer risk assessment tools) that often. That is something that I can’t fit into a 30-min visit, so if I (use them), it tends to be…after the visit. We might use the Gail model (or) the Tyrer-Cuzick model that’s more helpful with family history, but realistically am I doing that all the time in a 30-min visit? No.”
Awareness of B-PREP and patient clustering	“I think I personally am taking care of about 25 percent of all of the minority patients at (our clinic), so my lack of knowledge about (B-PREP) is probably one factor (contributing to lower referral rates). And I think many of our minority patients – Black and Latinx patients are cared for by our residents. And I'm not certain – you know, I think that if I hadn’t figured out that the program existed, I don't know how our residents would have known that it existed “I think that they – there’s a lack of awareness among many of the providers, it sounds like, taking care of the Black and Latinx women.” “At many centers like the Brigham where a higher percentage of the minority patients are taken care of by residents, we run the risk that every single one of the preventive measures our counseling measures are being done less actively for the minority patients.”
Confusion between B-PREP and genetics	“If there’s multiple family members (with cancer) that’s when (I will) talk about a genetics referral. It’s very confusing from our side when to refer to B-PREP versus genetics. I tend to use genetics more often, because we’re trying to figure out is there a genetic factor involved with a strong family history…I usually have to remind people multiple times to go.”
Limited value of B-PREP	“I don’t send (many) people to B-PREP because I feel like I have a pretty good understanding of this area.” “In my practice I would say, I’m not sure B-PREP is critical because I can offer a lot of what B-PREP does. And I think a really good PCP should be able to do that.”

*B-PREP* Breast Cancer Personalized Risk Assessment, Education and Prevention program, *USPSTF* United States Preventive Services Taskforce, *ACS* American Cancer Society, *BRCA* BReast CAncer gene, *NIH* National Institutes of Health

**Table 4:** Example clinician quotations regarding perceived contributors to disparities in B-PREP referrals

Contributors	Example quotes
Patient concern and discussions with patients	“The higher socioeconomic patients, which in my practice generally are White, are more attuned to breast cancer risk, more nervous about breast cancer, more aware of risk reduction and risk…do they need a MRI…do they want an ultrasound. That probably contributes to (who I refer).” “The people that I’ve sent to B-PREP are (those) that want more than I can give, and they…are doubting (me). Am I enough of an expert in this area to give them all the answers that they want? That (then) leads me to say: there’s this great clinic called B-PREP and they can answer your questions.” “It is not always feasible for underrepresented populations to be as assertive and to get the same outcome.” “Maybe through racism, providers are less likely to have the nuanced discussion (with people of color) that often precedes referring to B-PREP.” “I think, personally, doctors are more likely to dismiss minority patients’ concerns and see them as being angry or over advocating for themselves or being anxious or all sorts of demeaning ways of seeing it when they do advocate for themselves… I do think that communication problems between Black patients and Latinx patients with doctors who are different from them may contribute, or not, you know, like letting them talk enough or not listening to their concerns or focusing on other things and not making enough time to talk about these sorts of things.”
Awareness of breast cancer risk	“When it comes to these screening programs and risk modification programs, there is a perception that it’s just for White women.” “I think part of cultural and our American society has kind of steered the conversation toward breast cancer as more common in White women. Even though we know that’s not true, but that perception is out there.” “(For) White women, breast cancer is all those pink ribbon things – even though there’s been an attempt to change that,…the image is that breast cancer is for White women.”
Competing morbidities	“I think that competing priorities operate both for patients and for providers.” “Due to socioeconomic (factors) and structural racism, Black women are more likely to have more medical problems, so that the visit is like more tied up with that.” “The things that would make me more likely to refer someone would be if I actually took the time to talk to them and hear about their risk factors. And so, maybe there’s a difference in the kinds of conversations that doctors have with their patients depending on the patient’s race.”
Language barriers	“For my Spanish-speaking patients, they’re often more comfortable being seen at the health center. We have a lot of specialists who are available and come to our health center and that can be much easier geographically and logistically (for Spanish-speaking patients than going into Boston main campus for B-PREP).” “It is harder for me to have a nuanced discussion about risk in Spanish than it is in English…our phone interpreters are very variable and the time that you have to spend with a Spanish-speaking patient is the same as the time that you have with an English-speaking patient, however, the visit is literally one half as long because everything has to get said twice.”

*B-PREP* Breast Cancer Personalized Risk Assessment, Education and Prevention program, *MRI* magnetic resonance imaging

**Table 5:** Example clinician quotations regarding interventions to address referral inequities

Suggestions	
Increased education for primary care clinicians about B-PREP services and referral processes	“More education and tools that would help primary care physicians understand their patients’ risk of breast cancer, and support them in conversations with patients…along with that, understanding of B-PREP itself.” “Ongoing education to providers about the services is helpful and necessary, recognizing that we are at the end of a fire hose of information coming from all different places in primary care…sometimes we are not aware we could be under-referring, or we could be making inappropriate referrals.” “Clarifying the referral process…if we understood it better, we would refer better.”
Marketing beyond main hospital practices	“They can do a better job marketing themselves…within the Brigham Hospital system, periodically coming and visiting a practice, and saying, this is what we do.” “A marketing strategy that makes sure that at least all of the primary care practices know what’s there, and what added value that would be (for patients and providers).” “(Our Community Health Center) has a mammogram van every two months in our parking lot. I could imagine somebody from B-PREP coming here, and maybe not once every two months, but in association with that day.”
Strategies to make it easier for clinicians to assess risk	“If you could figure out a way to calculate a breast cancer risk score…on all patients, and then flag that information for doctors, and say: this is elevated and your patient might be a candidate for prevention, or increased surveillance testing, increased screening, or all (of the above)…click here for more information, or click here to refer your patient to the B-PREP clinic, (this would be helpful).” “You need to be supporting PCPs in doing cancer risk assessments on all their patients, not just patients who ask about it.”

*B-PREP* Breast Cancer Personalized Risk Assessment, Education and Prevention program, *PCPs* primary care providers

None of the clinicians interviewed described structured or systematic approaches to assessing breast cancer risk (Table 3). Most described discussing breast cancer risk with patients around age 40 in the context of discussions about timing and frequency of mammogram screening. In some cases, if patients brought up concern about breast cancer or if the patient had a strong family history of breast cancer, the conversation would start at a younger age. Risk assessment largely focused on family history, with little attention paid to other risk factors for breast cancer. Clinicians did not routinely use breast cancer risk assessment tools, though many expressed feelings that they should use them or would use them in an ideal practice setting if they had capacity and/or time to do so. Time was most consistently identified as a primary barrier.

2) Clinicians’ awareness and use of the B-PREP clinic was variable

Many clinicians expressed some confusion about what the B-PREP clinic offered and how it differed from a genetics clinic; several were more likely to refer to and be aware of genetics clinic (Table 4). One clinician reflected that patients of color were often “clustered” with a subset of PCPs that included several resident physicians; this clinician hypothesized that awareness of B-PREP could be lower among those residents caring for diverse patients. One clinician felt B-PREP did not have a lot of value above what she could offer in primary care. However, all who had referred patients to B-PREP described positive experiences.

3) Clinicians had varied perceptions of how race, ethnicity, and language might be associated with the extent to which breast cancer risk discussions are held with patients and whether referrals are made

Some clinicians noted that they referred patients to B-PREP when patients voiced concern about their breast cancer risk and had more questions than the primary care clinician could answer (Table 5). While some clinicians perceived that these concerns were brought up more by White women, others felt that breast cancer risk concerns brought up by women from minoritized racial and ethnic groups might simply be received differently by clinicians. Some participants perceived that clinicians may have lower awareness of breast cancer risk among minoritized groups and perceive breast cancer as a more pressing issue among White women; one clinician noted that Ashkenazi Jewish heritage could raise concern about a BRCA mutation. While a few participants felt that patients who were not White also might have lower risk awareness, more clinicians felt that Black and Hispanic patients for example, were no less concerned about breast cancer compared with White patients.

Structural barriers affected risk assessment and decisions about referrals. For example, language concordance and use of an interpreter could influence clinicians’ decision to have nuanced risk conversations. Several clinicians acknowledged that breast cancer risk assessment occurred less often for patients facing substantial socioeconomic barriers and medical comorbidities. One clinician at a community health center described substantial challenges faced by her largely Spanish-speaking patients with receiving specialty care at BWH, including getting appointments, receiving language concordant care, and understanding and trusting recommendations, and felt that anticipation of these challenges could lower clinicians’ likelihood of referring their patients to begin with.

4) Clinicians suggested health system-level interventions to address inequities in referral practices

Clinicians suggested increased marketing and education of the B-PREP program to primary care clinicians to increase awareness of the program and clarify the referral process. In addition to education about B-PREP to primary care clinicians, clinicians recommended outreach beyond the main hospital campus. Since time and knowledge of breast cancer risk assessment can be a barrier for primary care clinicians, there was a suggestion to make it easier for clinicians to calculate a risk score for all patients and for a prompt within the EHR system to appear if a patient is identified as high-risk and eligible for a B-PREP referral. In all interviews, primary care clinicians expressed the need for more education about B-PREP and a need to better understand which patients may benefit from referral.

## Discussion

This evaluation of care provided in an academic breast center found that among patients referred by primary care clinicians for breast health care, Black patients, those whose preferred language is not English, and publicly-insured patients were less likely to be referred for risk assessment than for evaluation of benign breast concerns relative to White, primarily English-speaking, and privately-insured patients, even when controlling for a patient’s underlying risk. Interviews showed that primary care clinicians lack a systematic approach to breast cancer risk assessment and referrals, which could provide opportunities for implicit bias in discussions. Primary care clinicians attributed inequities in referral patterns to a variety of factors, including barriers to risk discussions and misconceptions, particularly among clinicians, about breast cancer risk in diverse women.

These findings complement a limited body of evidence that suggests racial and ethnic inequities in conduct of breast cancer risk assessment and risk-stratified care [14–16]. In one study of 1,700 patients in California primary care practices, White women were more likely to have discussed genetic testing with their clinician and to have had genetic testing performed [14]. Among women with family history of breast and/or ovarian cancer, Black women were less likely to undergo *BRCA1/2* testing than White women [15]. In a study of 11 primary care practices in the greater Boston area, White and English-speaking women had a greater odds of being asked about family history of breast cancer by their primary care clinician compared to non-White and non-English speaking women regardless of age, education, or length of continuity with their primary care clinician [16].

Our findings have several implications for the B-PREP program. Recognizing the challenge of relying upon primary care clinicians to identify patients as high risk, the B-PREP program was deliberately designed to identify high-risk patients who may have been referred for another reason (for example, breast pain) by ensuring that all patients evaluated in the Breast Center complete a risk calculator and are referred to B-PREP if identified as high risk [17]. This practice may mitigate some of the referral inequities that we observed and could be a strategy that can be replicated in other breast centers. However, our findings still suggest that individuals without an indication for a BHC referral but who are high-risk may be under-referred, particularly members of groups that have been historically marginalized and experience worse breast cancer outcomes. Our findings about Black patients are particularly concerning since in the United States Black women face 40% higher breast cancer mortality relative to non-Hispanic White women [7]. Our findings strongly suggest that BWH and other institutions with specialized breast clinics need to engage groups that have been historically marginalized, as well as the primary care clinicians who care for them. Our study suggests several opportunities to accomplish this.

First, efforts to facilitate and standardize risk assessment in primary care could help ensure that risk assessment is equitably pursued. There are tremendous challenges with implementation of risk assessment in primary care, including identification of the most reliable and feasible tools, that will require dedicated efforts to overcome. Available risk calculators were developed and validated among White populations and ongoing work is underway to ensure that these calculators are accurate at predicting risk among diverse groups [23, 24]. Although risk calculators developed in Black women have been explored, [25] the notion that race should be considered in clinical calculators is highly contested since race is a social rather than biological construct [26, 27]. Within the field, single nucleotide polymorphisms and polygenetic risk score testing are being incorporated into models that include personal, family and lifestyle risk factors, and efforts are underway to validate these tools in diverse populations [23, 28–31]. Tools using artificial intelligence to determine risk from mammographic images are one promising strategy that does not incorporate race, but these tools are not yet in widespread practice [32, 33]. As tools are improved, strategies to facilitate implementation in primary care are essential, such as decision support integrated into an electronic medical record. Regardless of risk assessment tools used, providing patients from medically underserved racial and ethnic minority groups with breast cancer risk assessment and risk counseling has been shown influence their care and increase their likelihood of receiving mammography screening [34].

Second, primary care clinicians need to be aware of inequities in breast cancer care and outcomes and should be aware of the ways that both structural racism and bias may inform their approach to breast cancer risk. In the interviews, clinicians noted Ashkenazi Jewish ancestry as a criterion that influences their opinion on a patient’s breast cancer risk. Although this ancestry is associated with an increased risk of a pathogenic genetic variant in *BRCA1/BRCA2*, it is not the only pathogenic variant associated with Hereditary Breast and Ovarian Cancer Syndrome despite the emphasis it receives in practice guidelines [35, 36]. Since high-penetrance cancer genes are present among different racial and ethnic groups, some genetic experts and medical oncologists have advocated for expansion of genetic testing regardless of race, ethnicity and/or ancestry as criteria for testing [37]. Notably, the American College of Radiology released new guidelines recommending breast cancer risk assessment of all women by age 25 to determine if they should begin screening before age 40. These guidelines emphasize the importance of risk assessment in women who identify as Black/African American or of Ashkenazi Jewish ancestry [38]. In response to our findings, we plan more systematic efforts to increase awareness about the value of the B-PREP program among BWH primary care practices, and particularly those practices caring for diverse patient populations. Strategies to ensure the diversity of the clinicians who represent and practice in the program are being discussed. Community outreach could also increase patient awareness.

This project had several limitations. First, our study compared characteristics of patients referred to B-PREP for risk assessment to those referred to the BHC for benign concerns. We did not have breast cancer risk estimates for all women in BWH primary care practices over our study period, so we were not able to compare those referred to B-PREP to the underlying primary care population of women with the same estimated breast cancer risk. This could bias our conclusions – for example, if Black women were *more* likely than White women to be referred for benign breast concerns, our conclusions about lower rates of risk assessment referrals among Black women would be incorrect. Although we are not aware of any evidence to suggest racial or ethnic differences in prevalence of or care-seeking for breast symptoms, further study in larger populations will be important to extend this early work. Second, we had some missing data, including missing risk scores for patients who were referred and not scheduled or did not attend their appointment (Supplemental Table 4, Additional file 1). However, our findings were unchanged when we excluded those with unknown risk (Supplemental Table 2 in Additional file 1). Third, we did not have complete data on other important social determinants of health, such as socioeconomic status, which could impact referrals. In a sub-analysis of patients with zip code data for whom a SVI could be assigned, the inclusion of SVI did not reduce the impact of race, ethnicity, and preferred language on risk referral (Supplemental Table 1, Additional File 1) [39]. Fourth, our study does not include patients referred from primary care to the Genetics clinic, though given evidence of disparities in genetics consultations we believe it is unlikely that we would find that Genetics referrals would compensate for the disparities noted in referrals to B-PREP [15]. Finally, the findings of this single-site study are not necessarily generalizable to other medical facilities and populations, and should be further explored in a larger study and range of settings.

## Conclusions

In a single academic medical center, non-Hispanic Black individuals, publicly-insured individuals, and those whose preferred language is not English were less likely to be referred from primary care sites to a specialized clinic for breast cancer risk assessment. Lack of standardized risk assessment practices in primary care, misperceptions of risk, low familiarity with the B-PREP program, and structural barriers faced by patients may contribute to inequitable referral patterns. Further research from larger populations and other practices will be important to confirm these findings and their relevance to other settings. Education for primary care clinicians, systems to support equitable approaches to risk assessment, and enhanced accessibility of tailored risk assessment, risk-stratified screening, and risk reduction programs could improve quality of care for all.

### Supplementary Information

**Additional file 1.**

## Data Availability

The datasets used and/or analyzed during the current study are available from the corresponding author, upon reasonable request.
