# Supplementary material for: Inequities in referrals to a breast cancer risk assessment and prevention clinic: a mixed methods study
Source: BMC Prim Care. 2023 Aug 25;24:165. doi: 10.1186/s12875-023-02126-1 (PMC10464083; doi:10.1186/s12875-023-02126-1)
Supplement: Supplementary file 1 — Additional file 1. [file 12875_2023_2126_MOESM1_ESM.docx]

**Inequities in referrals to a breast cancer risk assessment
and prevention clinic: a mixed methods study**

**Additional File 1**

**Contains the following files:**

- **Supplemental Table 1:** Multivariable logistic regression subgroup analyses examining likelihood of referral for risk assessment among patients with known SVI (n=1,404)
- **Supplemental Table 2:** Multivariable logistic regression subgroup analyses examining likelihood of referral for risk assessment among patients with known risk level (n=1085)
- **Supplemental Table 3:** Characteristics of interview participants by primary care site type, gender identification, and self-identified race/ethnicity
- **Supplemental Table 4:** Demographics of patients with unknown risk and known risk with chi square tests
- **Supplemental Figure 1.** Pathways by which patients access services at the Brigham and Women’s Hospital Breast Cancer Risk Assessment, Education and Prevention (B-PREP) Program
- **Supplemental Figure 2:** Referral classification
- **Supplemental Figure 3:** Conceptual model
- **Exclusions (Figure 1)**
- **Referral Reason Classification**
- **Search Terms**
- **Referring Department identification**
- **Risk Scores**
- **References**

**Supplemental Table 1: Multivariable logistic regression subgroup analyses examining likelihood of referral for risk assessment among patients with known SVI (n=1,404)**

| Patient Demographics | Odds Ratio (95% CI) | P value |
| --- | --- | --- |
| **Race and Ethnicity**  Non-Hispanic White  Non-Hispanic Black  Hispanic  Asian American/Pacific Islander  Other  Unknown | Ref  **0.44 (0.25-0.78)**  0.67 (0.41-1.10)  0.81 (0.40-1.64)  0.74 (0.49-1.12)  0.41 (0.09-1.89) | **0.005**  0.11  0.56  0.15  0.25 |
| **Age**  Under 40  40-59  60+ | Ref  **0.64 (0.47-0.88)**  0.70 (0.47-1.04) | **0.006**  0.07 |
| **Preferred Language**  English  Non-English | Ref  **0.25 (0.10-0.61)** | **0.003** |
| **Risk Level***  Not High Risk  High Risk  Unknown Risk | Ref  **6.39 (4.29-9.52)**  **3.2 (2.19-4.73)** | **<0.001**  **<0.001** |
| **Insurance**  Commercial  Medicaid  Medicare  Other | Ref  **0.67 (0.47-0.96)**  0.59 (0.30-1.19)  0.76 (0.24-2.45) | **0.03**  0.14  0.65 |
| **Primary Care Site**  Suburban  Main Campus  Community Health Center  Other | Ref  1.21 (0.89-1.66)  1.31 (0.75-2.30)  0.60 (0.16-2.17) | 0.23  0.34  0.43 |

*High-risk was defined as patients having a 5-year modified Gail risk score of >1.67 for women between age 35-59, a 5-year modified Gail risk score of >5.5% for women age >60,(1) or a Tyrer-Cuzick v.7 or v.8 lifetime risk score >20%.(2)

**Supplemental Table 2: Multivariable logistic regression subgroup analyses examining likelihood of referral for risk assessment among patients with known risk level (n=1085)**

| Patient Demographics | Odds Ratio (95% CI) | P value |
| --- | --- | --- |
| **Race and Ethnicity**  Non-Hispanic White  Non-Hispanic Black  Hispanic  Asian American/Pacific Islander  Other  Unknown | Ref  **0.35 (0.17-0.72)**  0.92 (0.53-1.61)  0.81 (0.35-1.85)  0.92 (0.56-1.49)  0.36 (0.04-3.14) | **0.004**  0.78  0.62  0.72  0.36 |
| **Age**  Under 40  40-59  60+ | Ref  0.77 (0.53-1.14)  1.08 (0.66-1.77) | 0.19  0.75 |
| **Preferred Language**  English  Non-English | Ref  **0.26 (0.10-0.68)** | **0.01** |
| **Risk Level***  Not High Risk  High Risk | Ref  **7.8 (5.41-11.36)** | **<0.001** |
| **Insurance**  Commercial  Medicaid  Medicare  Other | Ref  0.78 (0.50-1.22)  0.86 (0.37-2.00)  0.24 (0.03-2.11) | 0.28  0.73  0.20 |
| **Primary Care Site**  Suburban  Main Campus  Community Health Center  Other | Ref  **1.69 (1.16-2.45)**  1.50 (0.84-2.70)  2.54 (0.64-10.14) | **0.01**  0.17  0.19 |

*High-risk was defined as patients having a 5-year modified Gail risk score of >1.67 for women between age 35-59, a 5-year modified Gail risk score of >5.5% for women age >60,(1) or a Tyrer-Cuzick v.7 or v.8 lifetime risk score >20%.(2)

**Supplemental Table 3: Characteristics of interview participants by primary care site type, gender identification, and self-identified race/ethnicity**

|  | Overall (%)  N=9 |
| --- | --- |
| **Primary care clinic site** |  |
| Main campus | 5 (55.6) |
| Suburban | 1 (11.1) |
| Community health center | 3 (33.3) |
| **Gender identification** |  |
| Woman/female/she series pronouns | 9 (100) |
| **Self-identified race/ ethnicity** |  |
| Non-Hispanic White | 6 (66.7) |
| Non-Hispanic Black/African American | 2 (22.2) |
| Non-Hispanic Other | 1 (11.1) |

**Supplemental Table 4: Demographics of patients with unknown risk and known risk with chi square tests**

| Patient Demographics | Unknown Risk (%)  (N=704) | Known Risk (%)  (N=1085) | P value |
| --- | --- | --- | --- |
| **Race/Ethnicity**  Non-Hispanic White  Non-Hispanic Black  Hispanic  Asian American/Pacific Islander  Other  Unknown | 367 (51.2)  79 (11.2)  141 (20.0)  20 (2.8)  84 (11.9)  13 (1.8) | 500 (46.1)  114 (10.5)  254 (23.4)  49 (4.5)  154 (14.2)  14 (1.3) | **0.05** |
| **Age**  Mean  Under 40  40-59  60+ | 48.7 (15.6)  227 (32.2)  298 (42.3)  179 (25.4) | 48.2 (13.8)  338 (31.2)  504 (46.5)  243 (22.4) | **0.18** |
| **Preferred Language**  English  Non-English  Unknown | 631 (89.6)  67 (9.5)  6 (0.9) | 933 (86.0)  149 (13.7)  3 (0.3) | **0.01** |
| **Insurance**  Medicaid  Medicare  Commercial  Other | 127 (18.0)  94 (13.4)  467 (66.3)  16 (2.3) | 353 (32.5)  45 (4.1)  673 (62.0)  14 (1.3) | **<0.001** |
| **Primary Care Site**  Main Campus  Community Health Center  Suburban  Other | 204 (29.0)  116 (16.5)  365 (51.8)  19 (2.7) | 373 (34.4)  205 (18.9)  493 (45.4)  14 (1.3) | **0.004** |

*High-risk was defined as patients having a 5-year modified Gail risk score of >1.67 for women between age 35-59, a 5-year modified Gail risk score of >5.5% for women age >60,(1) or a Tyrer-Cuzick v.7 or v.8 lifetime risk score >20%.(2)

Note: Of patients with an unknown risk score, 82.4% (580) were referred, but either never scheduled an appointment, or did not attend their scheduled appointment. The remaining 17.6% (124) attended an appointment but did not have a risk score generated due to an unknown reason.

**Supplemental Figure 1. Pathways by which patients access services at the Brigham and Women’s Hospital Breast Cancer Risk Assessment, Education and Prevention (B-PREP) Program**

**Supplemental Figure 2: Referral classification**


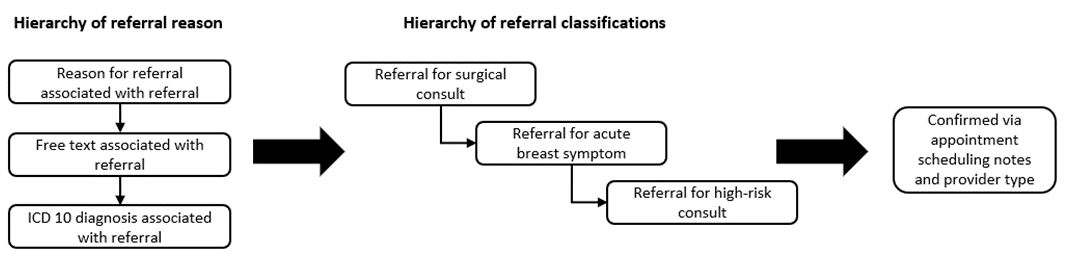


**Supplemental Figure 3: Conceptual model**


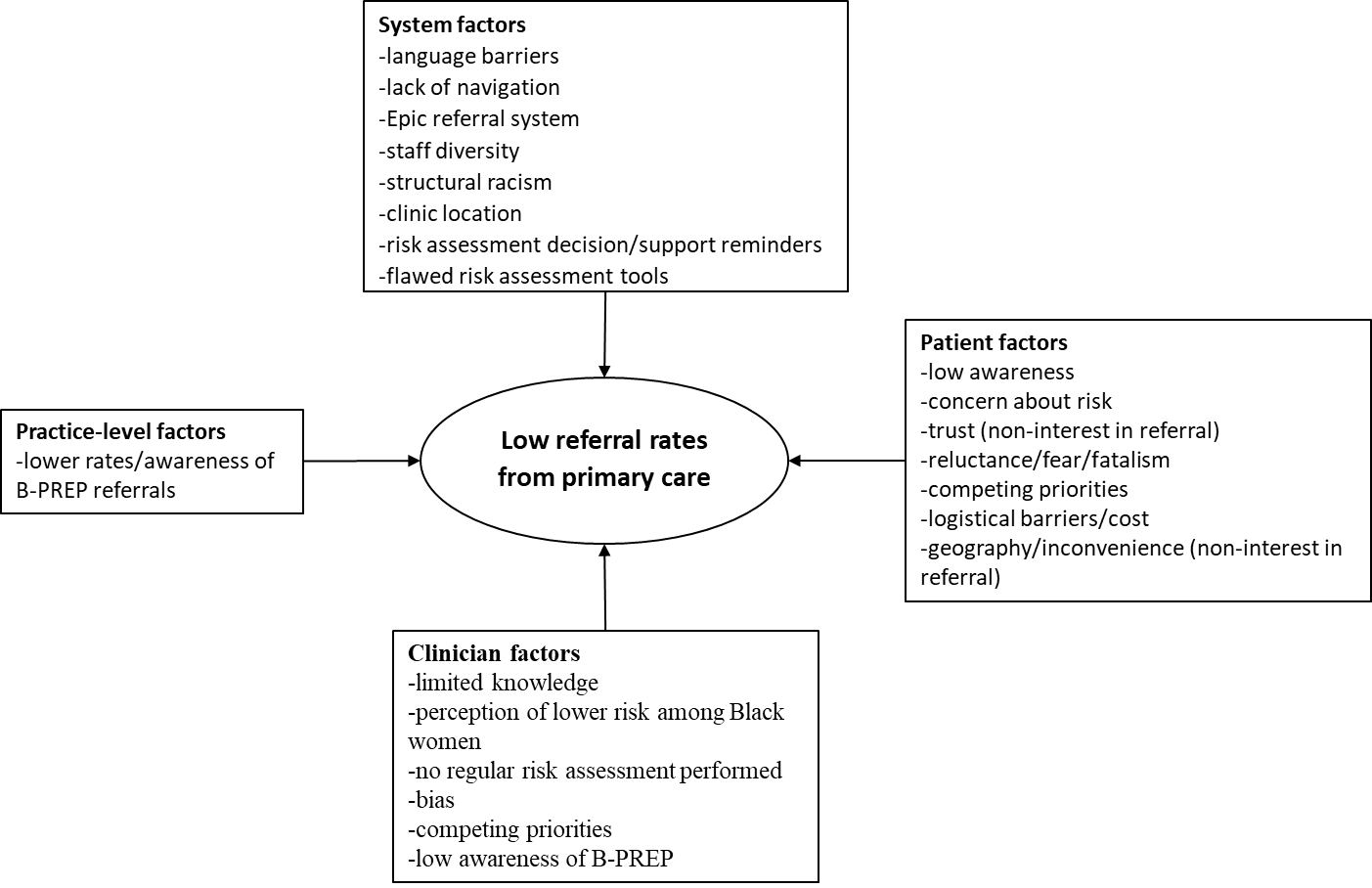


**Exclusions (Figure 1)**
*Established Patients*Data from completed appointments was retrieved from Epic EHR from January 2015 through December 2020 at both Brigham and Women’s Faulkner Breast Center and Brigham and Women’s Comprehensive Breast Center. There were 163 patients who completed a visit at the Comprehensive Breast Center prior to their referral date. Similarly, there were 172 patients who completed a visit at the Faulkner Breast Center prior to their referral date. These patients were excluded as they were presumed to be established patients within the clinic at the time of identified referral rather than a new patient to the clinic. There were 4,355 patients after this exclusion.

*Breast Cancer*

We excluded patients with a current diagnosis or personal history of breast cancer. There were 53 patients whose referral reason was “breast cancer”. Using ICD 10 codes attached to the referral as well as manual chart review, 33 additional patients were excluded due to having either a current or previous diagnosis of breast cancer. Using Hughes RiskApp data, an additional 49 patients who self-reported a history of breast cancer were excluded.

Manual review of free text referral reason and ICD-10 diagnosis code of remaining patients with unknown referral reason was performed and identified an additional 9 patients to exclude due to current or history of breast cancer.

*Patient Death*

Patient age was included in the referral report from Epic which identified 29 patients who were deceased at the time of data pull (July 2021). These patients were excluded.

*Plastic Surgery Referrals*

Using the referral report, patients were sorted into one of three reasons for referral: surgical, acute, or high risk. After this, there were 57 remaining patients with an unknown referral reason. The search terms, “implant”, “ruptured”, “reduction”, “augmentation” and “contracture” were used to identify patients who were referred for a plastic surgery reason. This identified 11 patients who were incorrectly referred to the breast center for a plastic surgery concern.

*Epic Test Patient*1 Epic HER test patient was removed.

*Referral from non-BWH Primary Care Sites*We excluded 2435 patients who were referred from a non-BWH site, or BWH site other than a primary care clinic. Our final cohort included only those patients referred to the Comprehensive Breast Center from a BWH-affiliated primary care clinic.

**Referral Reason Classification**

Initial referral reason classification was made using the reason for referral, free text associated with the referral, and ICD 10 diagnosis code attached to referral (See Supplemental Figure 3). Associated terms (see below) were used to match patients into Surgery, Acute, or High-Risk referral reasons. Referral reason priority was hierarchal: surgery, then acute, then high risk. For example, if a patient had a surgical and high-risk term match, they were sorted into the surgery bucket. After each of the 3 fields (the reason for referral, free text associated with the referral, and ICD 10 diagnosis code) were reviewed for referral classification, patients were sorted into their final bucket based on a combination of all 3. If “reason for referral” was known, that became their final classification. If “reason for referral” was unknown (as it was for many patients whose reason=add free text), then the free text classification was assigned. If both the reason for referral and free text classification was unknown, the ICD-10 classification was assigned. Only 30 patients in the final cohort remained with an unknown reason for referral. In our final cohort, we categorized patients referred for surgery or acute reason as referral for benign breast concern and those referred for high risk as referral to B-PREP for risk assessment.

Using only the above method, 379 patients were identified as referred to surgery. Appointment data from the Comprehensive Breast Center retrieved from Epic further refined reason for referral classifications. For patients identified as a surgery referral through information attached to the referral (reason for referral, free text reason for referral, and ICD 10 diagnosis), the clinician type of the patient's initial visit was identified. For patients classified as a surgery referral who did not see a surgeon (medical oncologist/NP/PA), they were sorted into the high-risk bucket. This was done to correct any patients that may have been referred for a risk discussion associated with a diagnosis of qualifying atypia that was already excised prior to their referral to the breast center. 252 of 379 patients were confirmed to either have seen a surgeon or did not have appointment data (if they had never scheduled an appointment) and were kept as referrals to surgery.

Based on clinic volume, we hypothesized that we were likely missing additional surgical patients. To determine if there were more surgical patients that were otherwise classified as acute or high risk due to lack of complete details in the referral, referral data for all patients was compared with breast center appointment data from January 2017 through June 15, 2021. Appointment scheduling notes were searched for the same list of surgery terms as well as “surgical consult”, which is routinely used by scheduling staff to identify surgical patients. The clinician for the corresponding visit was also identified and only those seen by a surgeon were classified as a surgical referral. The appointment note data identified 296 patients who were previously identified as acute, high risk, or unknown who were assigned as referred for surgery.

**Search terms*:**

Surgery Terms: "ADH", "atypical ductal hyperplasia", "FEA", "flat epithelial atypia", "flat epithielal", "Phyllodes", "phylloides", "fibroadenoma", "fiberoadenoma", "papilloma", "surgical", "CSL", "radial scar", "complex sclerosing", "variant LCIS", "pleomorphic", "PLCIS", "excisional", "excision", "sclerosing", "paget's", "paget", "prophylactic", "proph mastectomy", "prophylatic”

Acute Terms: "retraction", "pain", "discharge", "discahge", "thickening", "abnormal", "lump", "mass", "cyst", "abscess", "abcess", "absecess", "abscsess", "mastitis", "nipple", "inversion", "tenderness", "nodule", "lesions", "lesion", "macromastia", "rash", "cellulitis", "swelling", "discomfort", "tender", "discoloration", "abnl mammo", "itchiness", "asymmetry", "asymmetric", "aymmetry", "4B", "4A", "hardness", "infection", "calcification", "hematoma", "skin changes", "distortion", "arch dist", "nodular", "erythema", "duct", "pruruitis", "pruritus", "density", "nodularity", "galactorrhea", "milk", "fullness", "adenopathy", "palpable", "infection", "inflammation", "bleeding", "gynecomastia", "macromastia", "enlargement", "elargement", "macromastia", "gynecomastia", "fibromatosis", "amyloidosis", "galactocele", "PASH", "spindle cells", "mastopathy", "pseudo-angiomatous", "drainage", "dimpling", "atypical", "burning", "areolar", "indent", "mastodynia", "mastistis", "skin change", "benign", "ovoid", "fibroid", "lipoma", "skin", "accessorry breast tissue", "abnornmal mamo", "proliferation", "LN", "lymph node", "plaque", "ectopic", "breast size"

High Risk Terms: "family history", "family", "family hx", "fam hx", "family h/o", "fam h/o", "famil hx", "family hist", "famhx", "fmhx", "fm hx", "sister", "mother", "mom", "aunt", "grandmother", "MGM", "PMG", "dense", "risk", "LCIS", "lobular carcinoma in situ", "atypia", "ductal hyperplasia", "hyperplasia", "ALH", "genetic", "B-PREP", "FH", "BRCA1", "BRCA2", "BRCA", "CHEK2", "lynch", "maternal", "ashkenazi", "mutation", "screening", "cowden", "counseling", "prevention", "tyrer", "gail", "mily hx", "relatives", "radiation", "braca"

*Note: Some of the search terms are intentionally misspelled as that was how they were spelled in the referrals.

**Referring Department identification**

We chose to focus specifically our cohort on patients referred from Brigham affiliated primary care sites. These sites were identified using the Epic department code associated with each referral. We then grouped each BWH primary care site as a main campus (Jen Center, South Huntington, 800 Huntington, Brigham Circle Medical Associates), community health center (Brookside, Southern Jamaica Plain), suburban (850 Boylston, Family Care Brookline, Faulkner, Foxboro, HMA, Hyde Park, Newton, Norwood, PC Brookline, West Roxbury), or other BWH referring site.

**Risk Scores**

We matched breast cancer risk scores from B-PREP program customized survey adapted from Hughes RiskApps assessment data to referred patients using their BWH medical record number. Not all patients had risk scores available; if they were not seen at the clinic or did not complete the survey at their visit, then they did not have risk scores available. Patients were defined as high risk if they had Tyrer-Cuzick v.7 or v.8 lifetime risk score greater than 20%(2) or 5-year modified Gail risk score of >1.67 for women between age 35-59, a 5-year modified Gail risk score of >5.5% for women age >60.(1)

**References**

1. Gail MH, Brinton LA, Byar DP, Corle DK, Green SB, Schairer C, et al. Projecting individualized probabilities of developing breast cancer for white females who are being examined annually. J Natl Cancer Inst. 1989;81(24):1879-86.

2. Tyrer J, Duffy SW, Cuzick J. A breast cancer prediction model incorporating familial and personal risk factors. Stat Med. 2004;23(7):1111-30.
